# Precisely Molded Nanoparticle Displaying DENV-E Proteins Induces Robust Serotype-Specific Neutralizing Antibody Responses

**DOI:** 10.1371/journal.pntd.0005071

**Published:** 2016-10-20

**Authors:** Stefan W. Metz, Shaomin Tian, Gabriel Hoekstra, Xianwen Yi, Michelle Stone, Katie Horvath, Michael J. Miley, Joseph DeSimone, Chris J. Luft, Aravinda M. de Silva

**Affiliations:** 1 Department of Microbiology and Immunology, University of North Carolina, Chapel Hill, North Carolina, United States of America; 2 Lineberger Comprehensive Center, University of North Carolina, Chapel Hill, North Carolina, United States of America; 3 Department of Pharmacology, University of North Carolina, Chapel Hill, North Carolina, United States of America; 4 Eshelman School of Pharmacy, University of North Carolina, Chapel Hill, North Carolina, United States of America; 5 Department of Chemistry, University of North Carolina, Chapel Hill, North Carolina, United States of America; 6 Department of Chemical and Biomolecular Engineering, North Carolina State University, Raleigh, North Carolina, United States of America; 7 Sloan-Kettering Institute for Cancer Research, Memorial Sloan-Kettering Cancer Center, New York, New York, United States of America; 8 Liquidia Technologies, Research Triangle Park, North Carolina, United States of America; University of Texas Medical Branch, UNITED STATES

## Abstract

Dengue virus (DENV) is the causative agent of dengue fever and dengue hemorrhagic fever. The virus is endemic in over 120 countries, causing over 350 million infections per year. Dengue vaccine development is challenging because of the need to induce simultaneous protection against four antigenically distinct DENV serotypes and evidence that, under some conditions, vaccination can enhance disease due to specific immunity to the virus. While several live-attenuated tetravalent dengue virus vaccines display partial efficacy, it has been challenging to induce balanced protective immunity to all 4 serotypes. Instead of using whole-virus formulations, we are exploring the potentials for a particulate subunit vaccine, based on DENV E-protein displayed on nanoparticles that have been precisely molded using Particle Replication in Non-wetting Template (PRINT) technology. Here we describe immunization studies with a DENV2-nanoparticle vaccine candidate. The ectodomain of DENV2-E protein was expressed as a secreted recombinant protein (sRecE), purified and adsorbed to poly (lactic-co-glycolic acid) (PLGA) nanoparticles of different sizes and shape. We show that PRINT nanoparticle adsorbed sRecE without any adjuvant induces higher IgG titers and a more potent DENV2-specific neutralizing antibody response compared to the soluble sRecE protein alone. Antigen trafficking indicate that PRINT nanoparticle display of sRecE prolongs the bio-availability of the antigen in the draining lymph nodes by creating an antigen depot. Our results demonstrate that PRINT nanoparticles are a promising platform for delivering subunit vaccines against flaviviruses such as dengue and Zika.

## Author Summary

Dengue virus (DENV) is transmitted by mosquitoes and is endemic in over 120 countries, causing over 350 million infections yearly. Most infections are clinically unapparent, but under specific conditions, dengue can cause severe and lethal disease. DENV has 4 distinct serotypes and secondary DENV infections are associated with hemorrhagic fever and dengue shock syndrome. This enhancement of infection complicates vaccine development and makes it necessary to induce protective immunity against all 4 serotypes. Since whole virus vaccine candidates struggle to induce protective immunity, we are developing a nanoparticle display vaccine approach. We have expressed, purified and characterized a soluble recombinant E-protein (sRecE). Regardless of nanoparticle shape or size, particulation of sRecE enhances DENV specific IgG titers and induces a robust, long lasting neutralizing antibody response and by adsorbing sRecE to the nanoparticles, we prolong the exposure of sRecE to the immune system. Nanoparticle display shows great promise in dengue vaccine development and possibly other mosquito-borne viruses like zika virus.

## Introduction

Dengue virus (DENV), a member of the *flaviviridae* family, is the causative agent of dengue fever and dengue hemorrhagic fever. DENV and its *Aedes sp*. mosquito vectors are widely distributed in tropical and subtropical regions and is the most prevalent arthropod borne viral pathogen worldwide. Approximately half of the world’s population is at risk of being infected, resulting in up to 390 million reported cases of infection yearly. Roughly 1 million infections develop into severe disease of which nearly 2–5% is fatal [1,2]. More than 125 countries are endemic to DENV, but geographical expansion is expected to increase due to climate change, globalization of travel and trade and viral evolution [3–6]. Additionally, dengue is a complex disease resulting in a wide variety of clinical symptoms. The majority of infections are very mild or clinically in apparent. Infections are often misdiagnosed due to similarities between other prevalent tropical diseases. When symptoms are present, most patients undergo a sudden onset of fever that remains for 2–7 days, accompanied by arthralgia, myalgia and skin rash [7].

The dengue virus complex consists of 4 distinct serotypes designated DENV1-4. Primary infections induce long-term protective immunity to the serotype of infection only. Individuals are susceptible to secondary infections with a new serotype. Secondary heterotypic infections are associated with the more severe and potentially fatal dengue hemorrhagic fever or dengue shock syndrome [8]. As protective immunity to just one serotype may increase risk of disease upon exposure to other serotypes, leading dengue vaccines are based on tetravalent formulations to induce simultaneous immunity to all 4 serotypes. Several vaccine platforms are currently in preclinical or clinical development. These include live attenuated virus vaccines, live chimeric vaccines, inactivated virus formulations, recombinant virus vaccines, DNA and subunit vaccines [9]. Live virus formulations have progressed into clinical trials. The leading candidate, which has been tested in two large efficacy studies, demonstrated partial efficacy that varies between serotypes and based on the prior dengue exposure history of individuals [10]. Moreover, in some populations, the vaccine appeared to transiently increase risk of disease when people were exposed to a natural infection [10]. Recent advances in molecular biology have shown the safety and potential of rationally designed vaccine candidates, moving away from the whole virus paradigm and directing vaccinology towards subunit vaccine formulations. Subunit approaches for dengue generally involve the exploitation of E-protein as the major immunogen [11–14]. Even though several protein-based subunit vaccine approaches appear immunogenic and effective in small animals models, they have generally not been as immunogenic in primates [15].

The induction of an efficacious and protective immune response relies on proper antigen presentation to antigen presenting cells (APCs) including B cells, dendritic cells (DC) and macrophages, which are directly associated with production of antibodies and activation of T-lymphocytes and induce adaptive immunity leading to protective immunological memory. In general, subunit antigens as vaccine candidates are poorly effective as vaccine candidates due to size, degradation, non-specific targeting, the lack of cross-presentation and poor uptake by APCs. Coating soluble antigens to carriers is an effective strategy to increase the immunogenicity of weak immunogens. In the past decades, controlled release of prophylactic antigens has been achieved using nanoparticle vehicles or carriers composed of biodegradable polymers. One of the most promising aspects of coating subunit peptides to nanoparticle carriers is the potential to mimic structural and immunogenic features of the target pathogen. In addition to the generation of an antigen depot, the enhanced adjuvanticity of nanoparticles also contributes to enhanced vaccine efficacy by particulate antigen presentation and efficient targeting of APCs [16–19].

The pharmacokinetic characteristics of nanoparticles depend on the intrinsic properties of the particle itself. Particle size could have implications on APC uptake, biodistribution and bio-availability. Smaller particles are phagocytosed slower than larger particles, which contributes to better distribution by rapid dissolution [20]. Therefore, the optimization of nanoparticle composition, size and shape depends on disease target and application characteristics. One of the most commonly used polymeric nanoparticles are poly (lactic-co-glycolic acid) (PLGA) particles, a biodegradable polymer that can be tailored to vary degradability and release. A wide range of PLGA-based drug delivery systems have been used for the treatment and diagnosis of many diseases and disorders [21–23]. Many different fabrication processes have been utilized to produce PLGA based nanocarriers, out of which the PRINT (Particle Replication In Non-wetting Templates) process stands out in its capacity to control particle parameters [21,24–28]. The PRINT process creates monodispersed particles of uniform shape and size, is a scalable process and can be used to encapsulate or to be decorated with both hydrophilic and hydrophobic biological cargo [29–32].

Here we describe the results of studies to assess cationic PRINT PLGA nanoparticles of different size and shape as a platform for the delivery of a DENV2 E-protein (sRecE) antigen based vaccine. We demonstrate that PRINT nanoparticle adsorbed sRecE was maintained at the site of vaccination and within draining lymph nodes more efficiently than soluble antigen. The adsorbed antigen also induced higher IgG titers and DENV-2 specific neutralizing antibodies than soluble antigens.

## Material and Methods

### Cells and viruses

Vero cells were maintained as monolayer cell cultures at 37°C with 5% CO_2_, in DMEM (Gibco) media supplemented with 1% non-essential amino acids, 100 U/ml penicillin, 100 μg/ml streptomycin and 5% fetal bovine serum (FBS), which was lowered to 2% during infection.

EXPI293-cells were used for the production of sRecE and were maintained as a suspension culture in EXPI293 Expression Medium (Life Technologies). Cultures were passaged to 3×10^5^ cells/ml when cell densities of 3.5×10^6^ cells/ml were reached.

DENV1 WestPac-74, DENV2 S-16803, DENV3 CH53489 and DENV4 TVP-376 virus strains were used in the present study to determine antibody titers and in neutralization assays.

### Recombinant DENV2-E protein production and purification

The soluble recombinant DENV2 envelope protein (sRecE, aa1-395) was expressed using the EXPI293 transient expression system (ThermoFisher) using manufacturer supplied protocols. The N-terminal IL2 secretion leader peptide was fused to the DENV2 prM-sRecE cassette. sRecE was equipped with a C-terminal 6x histidine purification tag sequence (SSGGSHHHHHH) and expression was driven by a CAG (CMV early enhancer β actin) promoter. Supernatants were concentrated and buffer exchanged into a Ni^+2^ binding buffer (50mM NaPO_4_, 500mM NaCl, 25mM imidazole, 0.02% Na-Azide) via a tangential flow filtration and subjected to Ni^+2^ affinity chromatography. After washing with Ni^+2^ binding buffer, the Ni^+2^ column was step eluted with elution buffer (50mM NaPO_4_, 500mM NaCl, 500mM Imidazole 0.02%, Na-Azide, 10% glycerol). The fractions containing envelope protein were pooled and concentrated before being subjected to size exclusion chromatography using a 16/60 Superdex S200 column that was equilibrated with PBS containing 10% glycerol. Fractions with envelope protein were pooled, concentrated to 3mg/ml, flash frozen in liquid N_2_, and stored at -80°C. The resulting envelope protein was >95% pure as assessed by SDS-PAGE.

### Protein analysis

Purified sRecE protein fractions were subjected to sodium dodecyl sulphate polyacrylamide gel electrophoresis (SDS-PAGE) and analyzed by Western Blot (WB) and Coomassie Brilliant Blue (CBB) staining. 100 ng sRecE was resuspended and denatured in a gel loading buffer containing SDS. Following electrophoresis, proteins were transferred to a nitrocellulose membrane and blocked with 3% skim milk in TBS + 0.05% Tween-20 for 1 hr at RT. Next, membranes were subjected to 0.5 μg/ml 4G2 Mab in blocking buffer for 1 hr at 37°C. Membranes were washed 3 times with blocking buffer and treated with HRP-conjugated anti-mouse IgG (1:1000 in blocking buffer) for 1 hr at 37°C. After washing, the membranes were developed using ECL Prime Western Blotting Detection Reagent (Amersham).

### sRecE antigen capture ELISA to assess protein conformation

Ni^2+^-coated plates (Pierce) were coated with 100ng/well sRecE in TBS buffer (50 mM Tris, 150 mM NaCl, pH 7.5) for 1 hr at 37°C. The plates were washed (TBS+0.05% Tween-20) and blocked (washing buffer + 3% skim milk) for 1 hr at 37°C. The blocking buffer was discarded and the plates were incubated with 100 ng/well of the indicated mouse-derived (4G2, 3H5, DV2-46, 8A1) or human-derived (1M7, DVC-10.16, 2D22) Mabs in blocking buffer for 1 hr at 37°C. After incubation, plates were washed and treated with alkaline phosphatase (AP) conjugated α-human IgG (Sigma, 1:2500) or α-mouse IgG (Sigma, 1:1000) in blocking buffer for 45 min at 37°C. Plates were subsequently washed and developed using AP-substrate (Sigma). Absorbance was measured at 405 nm.

### Particle fabrication and characterization

The PRINT technology was employed to manufacture the monodisperse PLGA (50:50, 35 kDa, Lakeshore Biomaterials) particles as previously published [21,23]. Briefly, PLGA and DC-cholesterol (Avanti Polar lipids) were dissolved in chloroform (90:10 weight ratio) and casted into a thin film on a PET-sheet (KRS plastics). The film was then placed in contact with the patterned side of the molds and passed through a heated nip (ChemInstruments Hot Roll Laminator). The film was split and the filled mold was placed on second PET-sheet and subsequently passed through the laminator to transfer particles from mold to the PET-sheet. Next, the PET-sheet was incubated in water supplemented with 0.1 wt% polyvinyl alcohol (PVOH) to harvest particles. The particle suspension was sterilized using a 0.2 μm polyether sulfone membrane (Millipore) and purified by tangential flow filtration with a 0.05 μm polysulfone hollow fiber membrane (Spectrum Laboratories). The size and ζ-potential measurements were conducted on PLGA particle dispersions in 1 mM KCl solution using Zetasizer Nano ZS particle analyzer (Malvern Instruments) (S1 Table). Particle concentration was determined by thermogravimetric analysis (TGA) (TA Instruments). The actual loading level of DC-cholesterol was determined by reverse phase High-performance liquid chromatography (HPLC) with an Evaporative Light Scattering Detector (ELSD).

### sRecE adsorption to PLGA particles

sRecE (theoretical PI = 6.83) was incubated with NPs at indicated ratios for 15 min at RT in 0.1% polyvinyl alcohol (PVOH) in water. The particles were then pelleted by centrifugation for 15 min at 21000 × g. The amount of residual soluble protein was determined by a Quanti-iT Protein Assay Kit (Life Technologies) following manufacturer’s instructions. For immunizations in mice, the formulations also contained 9.25% sucrose to be isotonic (pH 7.4).

### Transmission electron microscopy

Discharged copper 400 mesh formvar carbon coated grids (Ted Pella Inc, Redding, CA, USA) were loaded with 10 μl PLGA particle solution for 5 mins at RT and washed 3 times in MilliQ water. The grids were stained 30 sec with 2% uranyl acetate. Excess uranyl acetate was removed and grids were air-dried at RT and observed with a LEO 910 transmission electron microscope (Zeiss).

### Mouse immunizations

Female Balb/c mice were purchased from Jackson Laboratory and used at age 6–12 weeks. All experiments involving the mice were carried out in accordance with an animal use protocol approved by the University of North Carolina Animal Care and Use Committee. Mice were euthanized by CO_2_ followed by cervical dislocation. Mice were immunized subcutaneously in the flank with 5 μg soluble (s)RecE (n = 4), sRecE+500 μg Alum (n = 4), PBS (Vehicle n = 3) or RecE adsorbed to 500 μg of PLGA/DC-chol (n = 5 per size; 80x320 nm, 80x180 nm, 55x70 nm and 200x200 nm) particles, on day 0, 21, and 63. All groups were immunized with same antigen dose, Serum samples were collected by submandibular bleeding on day 21, 28, 70, 98, 154, and 210.

### Evaluation of vaccine induced antibodies by IgG End Point Dilution Assay

ELISA plates were coated with 100 ng/well of IM7 Mab in 50 mM carbonate/bicarbonate buffer and incubated overnight at 4°C. The next day, the plates were washed in washing buffer (PBS with 0.05% Tween-20) and blocked with blocking buffer (washing buffer + 3% skim milk) for 1 hr at 37°C. Next, plates were loaded with DENV2 in blocking buffer and incubated for 1 hr at 37°C. After washing, the immunized mice sera was serially diluted in blocking buffer and loaded on the plates for 1 hr at 37°C. The plates were washed and subjected to AP-conjugated α-mouse IgG (Sigma, 1:1000) for 45 mins at 37°C and developed after washing with AP-substrate (Sigma). Absorbance was measured at 405 nm. The vehicle group was used to determine the background signal of the assay. The dilution where the sera from the experimental groups reaches background levels was calculated using GraphPad Prism software and used a measure of end point dilution titer.

### Evaluation of vaccine induced antibodies by DENV neutralization assay

We used a flow cytometry based neutralization assay to measure DENV neutralizing antibodies, which has been previously described in detail [33]. In brief, Vero cells were seeded in a 96-well culture plate at 2.5×10^4^ cells/well and incubated 24 hrs at 37°C. Mice sera were serially diluted in OptiMEM (Gibco) supplemented with 2% FBS. The appropriate amount of virus to establish a ~ 15% infection (determined previously) is added to the diluted sera and incubated 45 min at 37°C. Next, the cells are washed once with OptiMEM and the virus incubated with sera is added to the cells for 2 hr at 37°C. After incubation, the cells are washed once with growth medium and incubated overnight (ON) in 200 μl growth medium at 37°C. The next day, cells are washed with phosphate buffered saline (PBS) and treated with 0.05% trypsin (Gibco) for 10 min at 37°C. Detached cells were resuspended in PBS and transferred to a new 96-wells round-bottom culture plate and spun down at 1500 rpm for 5 mins. Cells were fixed in 4% paraformaldehyde and incubated for 10 min at room temperature (RT). Next, 150 μl permeabilization buffer was added to the fixed cells and spun down 5 min at 2500 rpm. Cells were washed once in perm buffer and spun down. Next, cells were blocked in permeabilization buffer supplemented with 1% normal mouse serum for 30 min at RT. Cells were subsequently incubated with Alexa fluor 488 conjugated anti-prM Mab 2H2, diluted 1:400 in blocking buffer for 1 hr at 37°C. Cells were washed in perm buffer and finally resuspended in FACS buffer. The percentage of infected cells was determined by flow cytometry using a Guava Flow Cytometer (EMD Millipore) and the neutralizing efficiency of the sera was expressed as neut_50_ values (the dilution where 50% of the virus was neutralized) calculated using GraphPad Prism software.

### Lymphatic drainage studies

RecE was labeled with fluorescent dye Alexa Fluor 647 using Alexa Fluor 647 Protein Labeling Kit (Life Technologies). Lymphatic drainage of antigen was examined as previously described [32]. Mice were injected subcutaneously in the rear right footpad with 2 μg RecE-Alexa Fluor 647 alone or adsorbed to 50 μg PLGA particles in 40 μL volume. Mice were sacrificed at 1, 6, 24, 48 or 72 hr post injections. Draining popliteal lymph nodes and footpads were resected, and imaged for total fluorescence using IVIS Lumina (PerkinElmer) imaging system with excitation at 640 nm and Cy5.5 emission filter. Analysis was done using Living Image software, version 3.2.

### Cell uptake of antigen by bone marrow derived dendritic cells (BMDCs)

Bone marrow was collected from mouse femurs and tibias as previously described [34]. Erythrocytes were lysed by NH_4_Cl. Bone marrow cells were subsequently cultured at 2x10^6^ cells/ml in RPMI 1640 (Gibco) supplemented with 10% FBS, 2 mM L-glutamine, 10 U/ml penicillin and 10 μg/ml streptomycin, 50 μM 2-mercaptoethanol, 10 ng/ml IL-4 and 10 ng/ml granulocyte—macrophage-colony stimulating factor (GM-CSF). The culture medium was replaced with fresh medium on day 3. BMDCs were harvested on day 6 and further purified with Opti-Prep density medium (Sigma) to remove dead cells. To test cellular uptake of antigen, RecE-Alexa Fluor 647 was mixed with PLGA particles in H_2_O and incubated at room temperature for 15 min, after which complete growth medium (RPMI 1640 with 10% FBS) was added. Day 6 purified BMDCs were dosed with samples for 24 hr at 37°C. Cells were washed twice with PBS, fixed with 4% paraformaldehyde for 10 min at RT. Next, cells then stained with 30 μM DAPI for 20 min at room temperature. Cells were washed, mounted with Fluorsave (EMD Millipore), and examined with a Zeiss 710 confocal microscope (Zeiss).

## Results

### Expression, characterization and adsorption of DENV2-sRecE to PLGA nanoparticles

**Fig 1:**
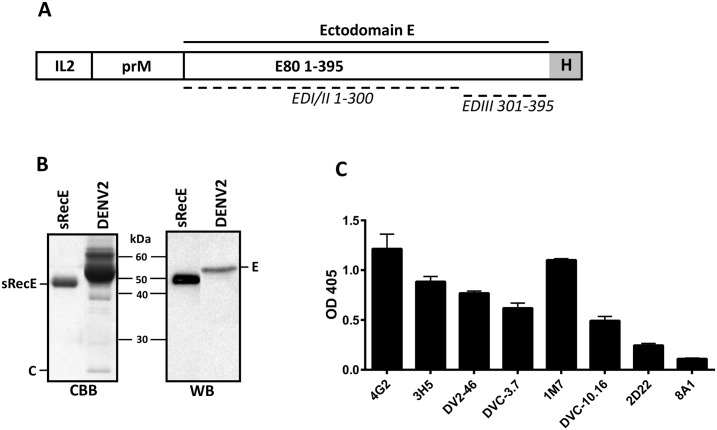
Expression and characterization of sRecE. **A)** Schematic representation of the sRecE expression construct. The DENV2 prM sequence leads the DENV2 E ectodomain (aa1-395) that is equipped with a C-terminal His-tag. The prM/sRecE is N-terminally fused to the IL2-leader sequence. **B)** Purified sRecE was subjected to SDS-PAGE and analyzed with CBB and WB using a 4G2 mouse derived Mab. **C)** Purifed sRecE was loaded on a Ni^2+^ coated ELISA plate and analyzed with a panel of mouse and human derived Mab. 8A1 is a DENV3 specific Mab and was used as a negative control.

The DENV2 (S-16803) full prM sequence and the ectodomain of E (aa1-395) with a C-terminal histidine tag was cloned downstream of an IL2 leader sequence (Fig 1A) in the pAH-expression vector. Secreted RecE (sRecE) was produced in EXPI293 cells and purified from the supernatant using His-affinity chromatography. Analysis of the final product by Coomassie Brilliant Blue (CBB) staining and Western blot (WB) detection with mouse Mab 4G2 showed high yields of purified recombinant protein (Fig 1B). sRecE had a slightly lower molecular weight (expected size of ~45kDa) then the full length wildtype E-protein control containing the membrane proximal and integral regions of the protein. To analyze sRecE structure and epitope accessibility, the His tagged protein was loaded on Ni^2+^-coated ELISA plates and detected using a panel of well-characterized DENV Mabs (Mouse derived: 4G2, 3H5, DV2-46, human derived: DVC3.7, 1M7, DVC-10.16, 2D22 and 8A1). All tested DENV2 reactive Mabs (Table 1) were able to bind sRecE (Fig 1C). The DENV-complex reactive envelope domain II (EDII) fusion loop binding Mabs (4G2 and DV2-46) and the envelope domain III (EDIII) binding Mabs 3H5, DVC3.7 and DVC10.16 all bound to sRecE. The highly neutralizing, DV2 specific human Mab 2D22, which binds to E protein dimer dependent quaternary epitope showed weak binding indicating that a small fraction of the protein may be present as E-protein homo-dimers. The DENV3 type-specific Mab 8A1 was used as a negative control. These results demonstrate that DENV2 sRecE is pure and displays conformational epitopes present on the monomer.

**Table 1:** Dengue specific monoclonal antibodies.

Mab	M/H	Binding	Neutralization *(W/M/S)*	Binding region	Binding DENV serotypes				Ref
					*DV1*	*DV2*	*DV3*	*DV4*	
***4G2***	M	F-CR	W.	DII FL	++	++	+++	+++	[35]
***3H5***	M	DV2	S/DV2	DIII LR	-	+++	-	-	[36]
***DV2-46***	M	DV2	M/DV2	DI and DII	-	++	-	-	[37]
***DVC-3*.*7***	H	DV2	S/DV2	DIII LR	-	++	-	-	[38]
***1M7***	H	D-CR	M.	DII	+++	++	+++	+++	[39]
***DVC-10*.*16***	H	D-C	S.	DIII AS	+	+++	+	+	[38]
***2D22***	H	DV2	S/DV2	DI/DII Q	-	++	-	-	[40]
***8A1***	M	DV3	S/DV3	DIII LR	-	-	+++	-	[41]

A panel of well-defined mouse or human (M/H) derived Mabs were used to characterize sRecE epitopes. flavivirus cross reactive (F-CR), dengue cross reactive (D-CR), dengue complex (D-C), weakly, moderately or strong (W/M/S) neutralizing, E-domain I, II, III (DI, DII, DIII), fusion loop (FL), lateral ridge (LR), A-strand (AS), quaternary (Q).

**Fig 2:**
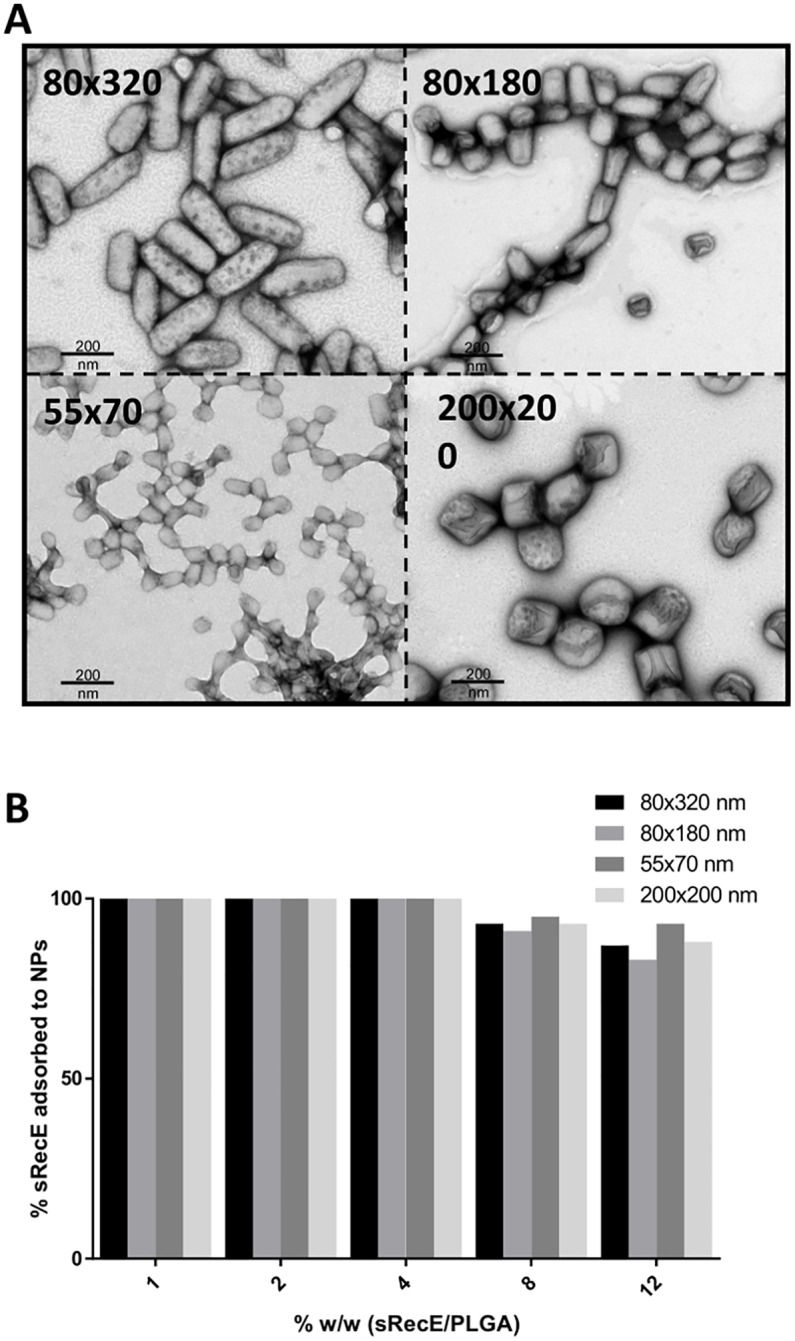
sRecE adsorption to PRINT PLGA nanoparticles. **A)** TEM analysis of 80×320 nm, 80×180 nm, 55×70 nm and 200×200 nm PLGA particles. **B)** sRecE was adsorbed to the PLGA nanoparticles in variable sRecE/PLGA (w/w%) ratios. Increasing sRecE/PLGA ratios were obtained by fixing sRecE and decreasing the particle mass. Adsorption efficiency was determined by the amount of non-adsorbed sRecE after the spinning down the particles.

Four different sized PLGA nanoparticles (80x320 nm, 80x180 nm, 55x70 nm and 200x200 nm) were produced using PRINT technology and analyzed by transmission electron microscopy (TEM) (Fig 2A). The TEM images showed a monodispersed particle distribution for each particle size. To adsorb sRecE to the PLGA nanoparticles, variable sRecE/PLGA (w/w%) ratios were mixed together for 15 minutes at RT, keeping the amount of sRecE constant and decreasing particle amount to obtain higher sRecE/PLGA ratios. The adsorption efficiency was determined by the amount of non-adsorbed sRecE after the spinning down the particles (Fig 2B). Up to a 4% w/w ratio resulted in nearly 100% adsorption efficiency for each particle size. The decrease in adsorption efficiency with increasing %w/w ratios (8% and 12%) was most likely caused by the excess of sRecE over the available particle surface.

### PLGA-RecE induces a durable neutralizing antibody response

**Fig 3:**
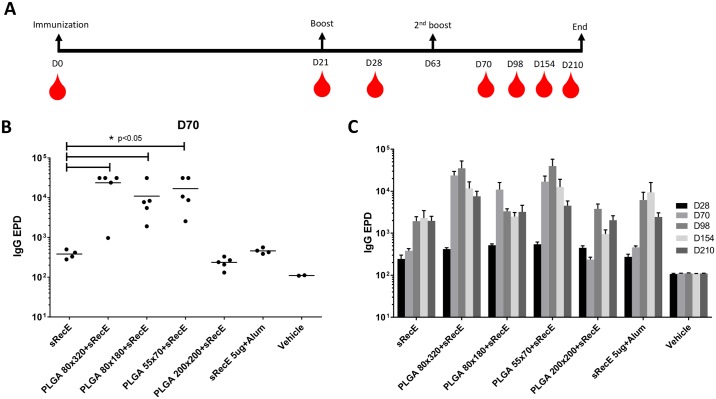
Long lasting IgG antibody titers induced by PLGA-RecE. **A)** Mice were immunized (subcutaneous) with 5 μg sRecE or 5 μg RecE adsorbed to PLGA particles. Animals were boosted with similar doses at day 21 and day 63 and mice were blead on indicated time points. **B-C)** DENV specific IgG end point dilution titers were determined at day 28, 70, 98, 154 and 210. Statistical differences were determined by one-way ANOVA followed by Tukeys test (p<0.05).

Mice were immunized (subcutaneous) with 5 μg sRecE or 5 μg RecE adsorbed to nanoparticles of indicated size. The animals were boosted twice on day 21 and 63 and serum samples were taken on day 0, 21, 28, 70, 98, 154 and 210. DENV2 specific end point dilution (EPD) IgG titers were determined on day 28, 70, 98, 154 and 210. On day 70 (one week post the second boost), the 80x320 nm, 80x180 nm and 55x70 nm PLGA+RecE particles induced significant higher DENV2 specific IgG titers compared to sRecE (Fig 3B). IgG titers induced by the 200x200 nm particle and adjuvanted sRecE (500 μg alum) were similar to those induced by sRecE. In general, IgG titers peaked at day 98 and remained elevated through day 210 (Fig 3C).

**Fig 4:**
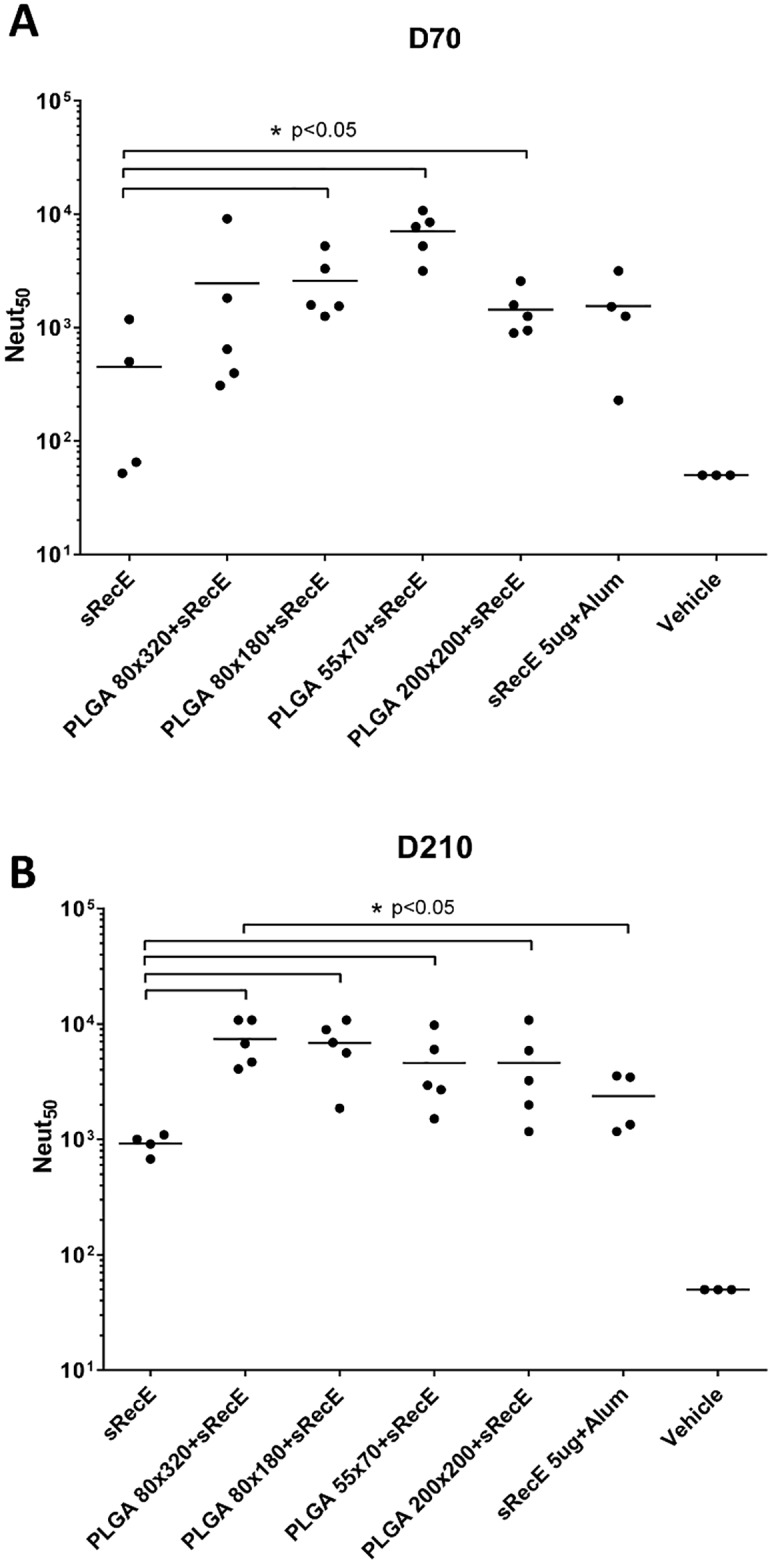
PLGA-RecE induces a long lasting neutralizing antibody response. The neutralizing activity of the mice sera was determined by a neutralization assay where DENV is incubated with serially diluted sera and subsequently allowed to infect Vero cells. Neutralizing activity was expressed as the dilution where 50% of the virus was neutralized (Neut_50_). **A)** At day 70 post immunization, 1 week post 2^nd^ boost and **B)** at day 210, 20 weeks post 2^nd^ boost. Statistical differences were determined by one-way ANOVA followed by Tukeys test (p<0.05).

The neutralizing activity of the sera collected at day 70 and day 210 was determined in a Vero-cell based neutralization assay and was expressed as the dilution where 50% (Neut_50_) of the virus was neutralized by antibodies in the sera. Mice immunized with the sRecE induced a mild neutralizing antibody response, which was increased with the addition of alum. At day 70 (Fig 4A), the 80x180, 55x70 and 200x200 PLGA particles decorated with sRecE induced significant higher neutralizing antibody titers than soluble sRecE. The PLGA 80x320+sRecE group showed differences between individual animals, but the group average was in the same order of magnitude as the other particulate groups. The high levels of neutralizing antibodies in the nanoparticle vaccinated animals was maintained through day 210 (Fig 4B), 21 weeks post 2^nd^ boost. These results demonstrate that sRecE adsorbed onto PRINT nanoparticles induced durable DENV2 neutralizing antibodies that were significantly higher than the levels maintained in animals that received the soluble antigen alone.

### sRecE induces a DENV2 specific neutralizing antibody response

**Fig 5:**
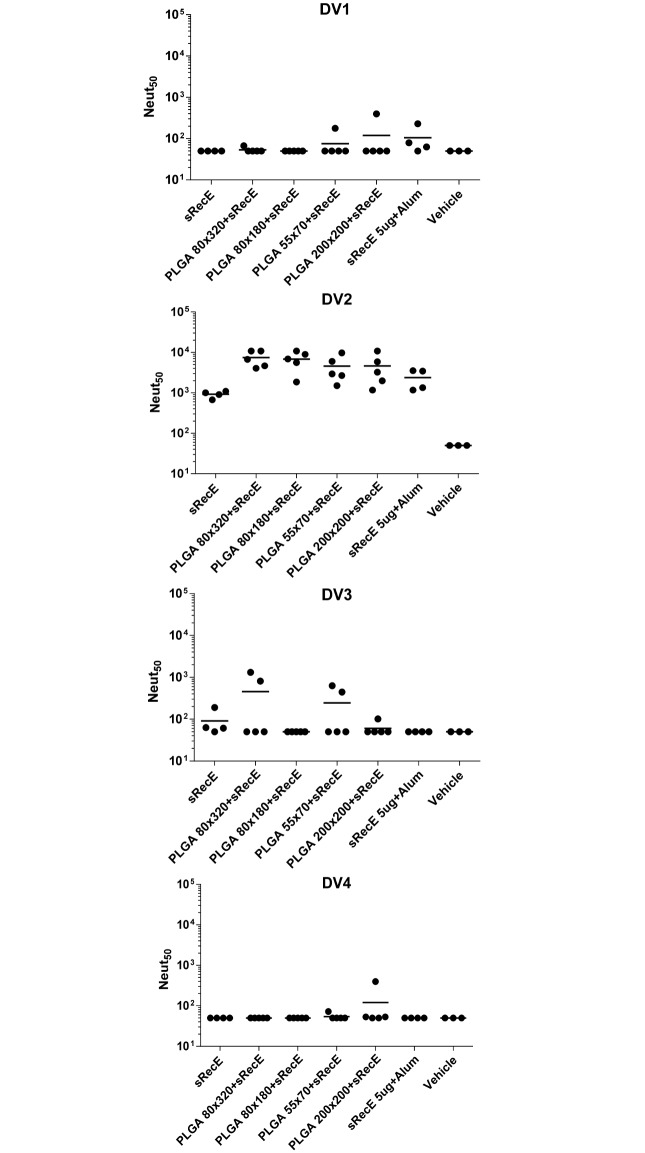
Cross-neutralization of RecE induced IgG antibodies. The capability of the cross reactive antibodies to neutralize DENV1, DENV2, DENV3 or DENV4 was tested in a neutralization assay using Vero cells. Neutralizing activity was expressed as the dilution where 50% of the virus was neutralized (Neut_50_).

The immune sera was analyzed for its ability to neutralize the other three DENV serotypes (Fig 5). Although the induced IgG response showed some mild cross-reactivity (S1 Fig), the neutralizing antibody response was strictly directed against DENV2 and showed no or poor cross-neutralization against the other DENV serotypes.

### sRecE adsorption increases bio-availability in the draining lymph nodes

**Fig 6:**
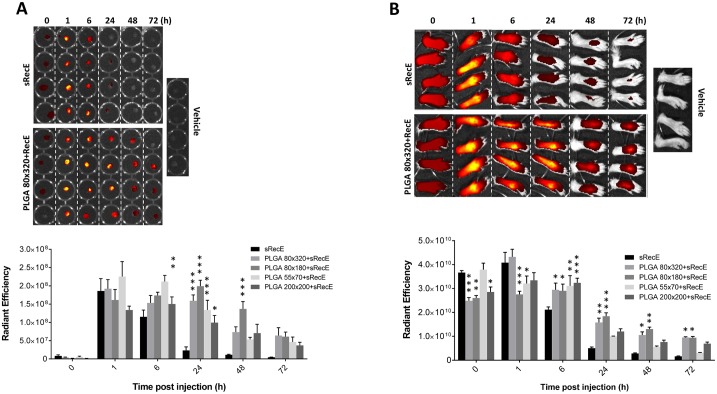
Bioavailability of sRecE and PLGA-RecE in PLNs and injection site. **A)** sRecE was tagged with Alexa Fluor 647 and adsorbed to PLGA nanoparticles and inoculated into to footpad. At indicated time points, the draining popliteal lymph nodes (PLNs) were isolated and analyzed for total fluorescence. The upper panel comparing sRecE and 80×320 nm PLGA-RecE is representative for the measure of antigen presence in the PLNs. The comparison of all nanoparticle sizes is depicted in the lower panel. **B)** The foot pads were analyzed at similar time points to analyze the bio-availability at the inoculation site. Statistical analysis was done by two-way ANOVA followed by Bonferroni posttests. * p<0.05, ** p<0.01, *** p<0.001.

We monitored the antigen in vaccinated animals to estimate bioavailability and efficiency of trafficking to lymph nodes. sRecE was fluorescently labeled and subsequently adsorbed to PLGA particles. After the indicated time points post footpad inoculation, the draining popliteal lymphnodes (PLNs) were isolated and analyzed for the presence of antigen (Fig 6A). Similar rapid initial drainage of antigen was observed for both sRecE and particle-adsorbed RecE at 1 hr post injections. However, the PLNs of mice inoculated with the sRecE mono-subunit was clearly detectable at 6 hr, yet undetectable at 24 hr post inoculation. The adsorption of sRecE to PLGA particles, regardless of size or shape, clearly increases bio-availability in the PLNs, since the antigen was still detected at 72hr post inoculation. Analysis of the inoculation site showed corresponding antigen presence, relative between the different groups (Fig 6B). Particulate sRecE was detected longer in the footpad compared to its soluble counterpart.

## Discussion

Yearly, over 300 million people are infected with DENV of which ~ 100 million symptomatic and 500,000 to 1 million develop severe disease resulting in ~25,000 deaths. Several groups are performing clinical trials with TV-live attenuated dengue vaccines. The leading candidate had lower efficacy against serotype 2 and also overall poor efficacy in young children [10], highlighting the importance of exploring new vaccine platforms. The use of nanotechnology in vaccine development has taken a rise in recent decades. Instead of using whole or live virus formulations to induce immunological protection against infectious diseases, subunit or subviral particle based platforms provide elegant and safe alternatives that have been proven efficacious against a large variety of viral pathogens [42–47].

In this study we explore the use of PLGA nanoparticles that are decorated with DENV2 E protein subunits. PRINT produced PLGA nanoparticles outperforms other nanoparticle production systems such emulsion, nanoprecipitation or spray-drying, in the control of particle shape and size uniformity [21,24–28]. The DENV-E protein is a large membrane-interface glycoprotein that regulates receptor recognition and viral fusion. In addition it is the major antigenic determinant for the development of a potent neutralizing antibody response. The purified sRecE antigen displayed conformational epitopes on EDII and EDIII targeted by human and mouse Mabs. Human Mab 2D22 binds to a quaternary structure epitope that is only displayed on E protein homo-dimers [48]. Mab 2D22 bound weakly to sRecE demonstrating the formation of some sRecE dimers.

Immunization with PLGA-RecE resulted in higher DENV IgG titers and DENV2 specific neutralizing antibodies than in animals vaccinated with the soluble antigen alone. At day 210 (the last time point tested) levels of neutralizing antibodies induced by PLGA-RecE were comparable to adjuvanted sRecE or on the case of 80x320 PLGA particles superior to adjuvanted sRecE. The inclusion of adjuvants in successive studies will reveal to what extend we can augment the PLGA-carrier capabilities.

Previous studies have demonstrated that size and shape of the nanocarrier influence processes such as cellular uptake and transport [20,49–51]. However, we did not observe a significant effect of PLGA particles size and shape on DENV E specific antibody responses. We did observe a trend of the 55 x 70 nm particles inducing higher levels of binding antibodies and the 80 x 320 nm particles inducing higher levels of neutralizing antibody although further studies are need to more precisely determine effects of particle properties on DENV immunogenicity.

In people exposed to primary DENV infections or monovalent vaccines, serotype-specific neutralizing antibodies have been implicated in durable protective immunity [8]. Therefore, we characterized specificity of the neutralizing antibody response to our monovalent (DENV2) vaccine antigen. Regardless of particle shape and size, PLGA-RecE induced neutralizing antibodies that were mainly DENV2 specific, indicating that the neutralizing response was targeted to unique epitopes on DENV2. Next we will directly test the efficacy of the PGA vaccine in mouse models of DENV infection and disease In these follow-up challenge studies, we will look more closely into the properties of the neutralizing antibodies, such as epitope specificity, antibody affinity and IgG isotype. We will also analyze the breadth of the response by testing if different genotypes of DENV2 are neutralized by the vaccine. Studies are also planned to test if tetravalent mixtures of PLGA-RecE can be used to induce a balanced and type-specific neutralizing responses to all 4 serotypes.

To better understand the mechanism responsible for the increased immunogenicity of the PLGA groups, we analyzed antigen uptake by mouse bone marrow derived dendritic cells (BMDCs), and followed the drainage of antigen from the footpad inoculation site to the PLNs. *In vitro*, both sRecE and PLGA adsorbed RecE were readily taken up by BMDCs (S2 Fig), indicating that uptake into these antigen presenting cells was unlikely to be responsible for the enhanced immunogenicity of the PLGA vaccine. PLGA adsorbed sRecE was detected at the site of vaccination and in the draining lymph nodes for much longer than sRecE alone indicating that extended bio-availability was most likely responsible for the enhanced antibody responses in PLGA vaccinated animals. We propose that PLGA vaccination leads to the formation of an antigen depot at the inoculation site and gradual release of antigen, whereas the soluble antigen alone is efficiently cleared after vaccination.

In this study we show that PRINT produced PLGA particles decorated with DENV2 sRecE are an effective platform to increase immune responses targeted against sRecE even in the absence of any adjuvant. Using this strategy we were able to induce long lasting serotype specific neutralizing antibody responses by increasing the bio-availability of the antigen. We clearly show the added effect of particulation on raised immune responses and antigen trafficking. Though only focusing on DENV2, these findings form the basis of further studies towards the other serotypes and might form the basis of a safe and efficacious dengue virus candidate. In addition, this platform can be used to develop safe vaccine candidates for other flaviviruses such as Zika virus, where pregnant women are the target group for vaccination.

## Supporting Information

S1 Table Physicochemical characterization of PLGA particles. PRINT produced PLGA nanoparticles of indicated sizes were examined by Nano zetasizer for dynamic light scattering (DLS), poly dispersity index (PDI) and Zeta potential.
(DOCX)

S1 Fig Cross-reactivity of sRecE induced IgG antibodies. The capability of the cross reactive antibodies (at week 30 post immunization) to detect DENV1, DENV2, DENV3 or DENV4 was tested by a DENV specific capture ELISA.
(TIF)

S2 Fig sRecE uptake by BMDCs. sRecE was tagged with Alexa Fluor 647 and adsorbed to PLGA nanoparticles. Bone marrow derived dendritic cells were mixed with the particles and analyzed with fluorescence microscopy to determine antigen uptake.
(TIF)

## References

[1] Bhatt S, Gething PW, Brady OJ, Messina JP, Farlow AW, et al (2013) The global distribution and burden of dengue. Nature 496: 504–507. 10.1038/nature12060 23563266 PMC3651993

[2] Thomas SJ, Endy TP (2011) Critical issues in dengue vaccine development. Curr Opin Infect Dis 24: 442–450. 10.1097/QCO.0b013e32834a1b0b 21799408

[3] Wilder-Smith A, Ooi EE, Vasudevan SG, Gubler DJ (2010) Update on dengue: epidemiology, virus evolution, antiviral drugs, and vaccine development. Curr Infect Dis Rep 12: 157–164. 10.1007/s11908-010-0102-7 21308524

[4] Wilder-Smith A, Gubler DJ (2008) Geographic expansion of dengue: the impact of international travel. Med Clin North Am 92: 1377–1390, x 10.1016/j.mcna.2008.07.002 19061757

[5] Astrom C, Rocklov J, Hales S, Beguin A, Louis V, et al (2012) Potential distribution of dengue fever under scenarios of climate change and economic development. Ecohealth 9: 448–454. 10.1007/s10393-012-0808-0 23408100

[6] Hales S, de Wet N, Maindonald J, Woodward A (2002) Potential effect of population and climate changes on global distribution of dengue fever: an empirical model. Lancet 360: 830–834. 10.1016/S0140-6736(02)09964-6 12243917

[7] Rigau-Perez JG, Clark GG, Gubler DJ, Reiter P, Sanders EJ, et al (1998) Dengue and dengue haemorrhagic fever. Lancet 352: 971–977. 10.1016/S0140-6736(97)12483-7 9752834

[8] Flipse J, Smit JM (2015) The Complexity of a Dengue Vaccine: A Review of the Human Antibody Response. PLoS Negl Trop Dis 9: e0003749 10.1371/journal.pntd.0003749 26065421 PMC4465930

[9] Wan SW, Lin CF, Wang S, Chen YH, Yeh TM, et al (2013) Current progress in dengue vaccines. J Biomed Sci 20: 37 10.1186/1423-0127-20-37 23758699 PMC3686670

[10] Halstead SB, Russell PK (2016) Protective and immunological behavior of chimeric yellow fever dengue vaccine. Vaccine: In press. 10.1016/j.vaccine.2016.02.004 26873054

[11] Guzman MG, Rodriguez R, Rodriguez R, Hermida L, Alvarez M, et al (2003) Induction of neutralizing antibodies and partial protection from viral challenge in Macaca fascicularis immunized with recombinant dengue 4 virus envelope glycoprotein expressed in Pichia pastoris. Am J Trop Med Hyg 69: 129–134. 13677367

[12] Kelly EP, Greene JJ, King AD, Innis BL (2000) Purified dengue 2 virus envelope glycoprotein aggregates produced by baculovirus are immunogenic in mice. Vaccine 18: 2549–2559. 10.1016/S0264-410X(00)00032-3 10775789

[13] Clements DE, Coller BA, Lieberman MM, Ogata S, Wang G, et al (2010) Development of a recombinant tetravalent dengue virus vaccine: immunogenicity and efficacy studies in mice and monkeys. Vaccine 28: 2705–2715. 10.1016/j.vaccine.2010.01.022 20097152 PMC2837772

[14] Coller BA, Clements DE, Bett AJ, Sagar SL, Ter Meulen JH (2011) The development of recombinant subunit envelope-based vaccines to protect against dengue virus induced disease. Vaccine 29: 7267–7275. 10.1016/j.vaccine.2011.07.021 21777637 PMC3179979

[15] Mamo T, Poland GA (2012) Nanovaccinology: the next generation of vaccines meets 21st century materials science and engineering. Vaccine 30: 6609–6611. 10.1016/j.vaccine.2012.08.023 23067445

[16] Bachmann MF, Jennings GT (2010) Vaccine delivery: a matter of size, geometry, kinetics and molecular patterns. Nat Rev Immunol 10: 787–796. 10.1038/nri2868 20948547

[17] Reddy ST, Swartz MA, Hubbell JA (2006) Targeting dendritic cells with biomaterials: developing the next generation of vaccines. Trends Immunol 27: 573–579. 10.1016/j.it.2006.10.005 17049307

[18] Storni T, Kundig TM, Senti G, Johansen P (2005) Immunity in response to particulate antigen-delivery systems. Adv Drug Deliv Rev 57: 333–355. 10.1016/j.addr.2004.09.008 15560945

[19] Zolnik BS, Gonzalez-Fernandez A, Sadrieh N, Dobrovolskaia MA (2010) Nanoparticles and the immune system. Endocrinology 151: 458–465. 10.1210/en.2009-1082 20016026 PMC2817614

[20] Zazo H, Colino CI, Lanao JM (2016) Current applications of nanoparticles in infectious diseases. J Control Release 224: 86–102. 10.1016/j.jconrel.2016.01.008 26772877

[21] Enlow EM, Luft JC, Napier ME, DeSimone JM (2011) Potent engineered PLGA nanoparticles by virtue of exceptionally high chemotherapeutic loadings. Nano Lett 11: 808–813. 10.1021/nl104117p 21265552 PMC3122105

[22] Hasan W, Chu K, Gullapalli A, Dunn SS, Enlow EM, et al (2012) Delivery of multiple siRNAs using lipid-coated PLGA nanoparticles for treatment of prostate cancer. Nano Lett 12: 287–292. 10.1021/nl2035354 22165988 PMC3358784

[23] Galloway AL, Murphy A, DeSimone JM, Di J, Herrmann JP, et al (2013) Development of a nanoparticle-based influenza vaccine using the PRINT technology. Nanomedicine 9: 523–531. 10.1016/j.nano.2012.11.001 23178283

[24] Liggins RT, Burt HM (2001) Paclitaxel loaded poly(L-lactic acid) microspheres: properties of microspheres made with low molecular weight polymers. Int J Pharm 222: 19–33. 10.1016/S0378-5173(01)00690-1 11404029

[25] Chung YM, Simmons KL, Gutowska A, Jeong B (2002) Sol-gel transition temperature of PLGA-g-PEG aqueous solutions. Biomacromolecules 3: 511–516. 10.1021/bm0156431 12005522

[26] Oster CG, Kissel T (2005) Comparative study of DNA encapsulation into PLGA microparticles using modified double emulsion methods and spray drying techniques. J Microencapsul 22: 235–244. 10.1080/02652040500100295 16019909

[27] Ehrenfried LM, Patel MH, Cameron RE (2008) The effect of tri-calcium phosphate (TCP) addition on the degradation of polylactide-co-glycolide (PLGA). J Mater Sci Mater Med 19: 459–466. 10.1007/s10856-006-0061-6 17607516

[28] Holgado MA, Arias JL, Cozar MJ, Alvarez-Fuentes J, Ganan-Calvo AM, et al (2008) Synthesis of lidocaine-loaded PLGA microparticles by flow focusing. Effects on drug loading and release properties. Int J Pharm 358: 27–35. 10.1016/j.ijpharm.2008.02.012 18372128

[29] Maynor BW, Euliss LE, Rolland LP, DeSimone JM (2005) Rational fabrication of polymeric nanostructures using pattern replication in non-wetting templates (PRINT). Abstracts of Papers of the American Chemical Society 230: U4049–U4049.

[30] Petros RA, Ropp PA, DeSimone JM (2008) Reductively labile PRINT particles for the delivery of doxorubicin to HeLa cells. Journal of the American Chemical Society 130: 5008–+. 10.1021/ja801436j 18355010

[31] Merkel TJ, Chen K, Jones SW, Pandya AA, Tian SM, et al (2012) The effect of particle size on the biodistribution of low-modulus hydrogel PRINT particles. Journal of Controlled Release 162: 37–44. 10.1016/j.jconrel.2012.06.009 22705460 PMC3416965

[32] Mueller SN, Tian S, DeSimone JM (2015) Rapid and Persistent Delivery of Antigen by Lymph Node Targeting PRINT Nanoparticle Vaccine Carrier To Promote Humoral Immunity. Mol Pharm 12: 1356–1365. 10.1021/mp500589c 25817072 PMC4545241

[33] Kraus AA, Messer W, Haymore LB, de Silva AM (2007) Comparison of plaque- and flow cytometry-based methods for measuring dengue virus neutralization. J Clin Microbiol 45: 3777–3780. 10.1128/JCM.00827-07 17804661 PMC2168473

[34] Madaan A, Verma R, Singh AT, Jain SK, Jaggi M (2014) A stepwise procedure for isolation of murine bone marrow and generation of dendritic cells. Journal of Biological Methods 1: e1 10.14440/jbm.2014.12

[35] Henchal EA, McCown JM, Burke DS, Seguin MC, Brandt WE (1985) Epitopic analysis of antigenic determinants on the surface of dengue-2 virions using monoclonal antibodies. Am J Trop Med Hyg 34: 162–169. 2578750 10.4269/ajtmh.1985.34.162

[36] Pitcher TJ, Sarathy VV, Matsui K, Gromowski GD, Huang CY, et al (2015) Functional analysis of dengue virus (DENV) type 2 envelope protein domain 3 type-specific and DENV complex-reactive critical epitope residues. J Gen Virol 96: 288–293. 10.1099/vir.0.070813-0 25351518 PMC4298678

[37] Sukupolvi-Petty S, Austin SK, Engle M, Brien JD, Dowd KA, et al (2010) Structure and function analysis of therapeutic monoclonal antibodies against dengue virus type 2. J Virol 84: 9227–9239. 10.1128/JVI.01087-10 20592088 PMC2937608

[38] de Alwis R, Beltramello M, Messer WB, Sukupolvi-Petty S, Wahala WM, et al (2011) In-depth analysis of the antibody response of individuals exposed to primary dengue virus infection. PLoS Negl Trop Dis 5: e1188 10.1371/journal.pntd.0001188 21713020 PMC3119640

[39] Smith SA, de Alwis AR, Kose N, Jadi RS, de Silva AM, et al (2014) Isolation of dengue virus-specific memory B cells with live virus antigen from human subjects following natural infection reveals the presence of diverse novel functional groups of antibody clones. J Virol 88: 12233–12241. 10.1128/JVI.00247-14 25100837 PMC4248927

[40] Fibriansah G, Ibarra KD, Ng TS, Smith SA, Tan JL, et al (2015) DENGUE VIRUS. Cryo-EM structure of an antibody that neutralizes dengue virus type 2 by locking E protein dimers. Science 349: 88–91. 10.1126/science.aaa8651 26138979 PMC4672004

[41] Zhou Y, Austin SK, Fremont DH, Yount BL, Huynh JP, et al (2013) The mechanism of differential neutralization of dengue serotype 3 strains by monoclonal antibody 8A1. Virology 439: 57–64. 10.1016/j.virol.2013.01.022 23453578 PMC3608513

[42] Schiller JT, Lowy DR (2015) Raising expectations for subunit vaccine. J Infect Dis 211: 1373–1375. 10.1093/infdis/jiu648 25420478 PMC4400525

[43] Zhang N, Tang J, Lu L, Jiang S, Du L (2015) Receptor-binding domain-based subunit vaccines against MERS-CoV. Virus Res 202: 151–159. 10.1016/j.virusres.2014.11.013 25445336 PMC4439384

[44] Tan M, Jiang X (2014) Subviral particle as vaccine and vaccine platform. Curr Opin Virol 6: 24–33. 10.1016/j.coviro.2014.02.009 24662314 PMC4072748

[45] Metz SW, Geertsema C, Martina BE, Andrade P, Heldens JG, et al (2011) Functional processing and secretion of chikungunya virus E1 and E2 glycoproteins in insect cells. Virol J 8: 353–365. 10.1186/1743-422X-8-353 21762510 PMC3162542

[46] Metz SW, Martina BE, van den Doel P, Geertsema C, Osterhaus AD, et al (2013) Chikungunya virus-like particles are more immunogenic in a lethal AG129 mouse model compared to glycoprotein E1 or E2 subunits. Vaccine 31: 6092–6096. 10.1016/j.vaccine.2013.09.045 24099875

[47] Metz SW, Pijlman GP (2011) Arbovirus vaccines; opportunities for the baculovirus-insect cell expression system. J Invert Pathol 107 Suppl: S16–30. 10.1016/j.jip.2011.05.002 21784227

[48] Gallichotte EN, Widman DG, Yount BL, Wahala WM, Durbin A, et al (2015) A new quaternary structure epitope on dengue virus serotype 2 is the target of durable type-specific neutralizing antibodies. MBio 6: e01461–01415. 10.1128/mBio.01461-15 26463165 PMC4620467

[49] Arvizo RR, Bhattacharyya S, Kudgus RA, Giri K, Bhattacharya R, et al (2012) Intrinsic therapeutic applications of noble metal nanoparticles: past, present and future. Chem Soc Rev 41: 2943–2970. 10.1039/c2cs15355f 22388295 PMC3346960

[50] Patel PA, Patravale VB (2011) AmbiOnp: solid lipid nanoparticles of amphotericin B for oral administration. J Biomed Nanotechnol 7: 632–639. 10.1166/jbn.2011.1332 22195480

[51] Tiwari S, Verma SK, Agrawal GP, Vyas SP (2011) Viral protein complexed liposomes for intranasal delivery of hepatitis B surface antigen. Int J Pharm 413: 211–219. 10.1016/j.ijpharm.2011.04.029 21540094

