# Supplementary material for: Precisely Molded Nanoparticle Displaying DENV-E Proteins Induces Robust Serotype-Specific Neutralizing Antibody Responses
Source: PLoS Negl Trop Dis. 2016 Oct 20;10(10):e0005071. doi: 10.1371/journal.pntd.0005071 (PMC5072622; doi:10.1371/journal.pntd.0005071)
Supplement: S1 Table — PRINT produced PLGA nanoparticles of indicated sizes were examined by Nano zetasizer for dynamic light scattering (DLS), poly dispersity index (PDI) and Zeta potential. (DOCX) [file pntd.0005071.s001.docx]

**Supporting information**

**STable 1: PLGA particle characteristics.**

| **Particle size** | **DLS (nm)** | **PDI** | **Zeta Potential (mV)** |
| --- | --- | --- | --- |
| 80x320 nm | 169 | 0.07 | + 28 |
| 80x180 nm | 139 | 0.01 | + 34 |
| 55x70 nm | 151 | 0.11 | + 22 |
| 200x200 nm | 201 | 0.01 | + 32 |

**SMethods:**

**Cell uptake of antigen by BMDCs.**

Bone marrow was collected from mouse femurs and tibias. Erythrocytes were lysed by ammonium chloride treatment. Bone marrow cells were then cultured at 2x10^6^/ml in RPMI 1640 supplemented with 10% FBS, 2 mM L-glutamine, 10 U/ml penicillin and 10 µg/ml streptomycin, 50 µM 2-mercaptoethanol, 10 ng/ml each of IL-4 and granulocyte–macrophage-colony stimulating factor (GM-CSF). The culture was replenished with fresh medium on day 3. BMDCs were harvested on day 6 and further purified with Opti-Prep density medium (Sigma) to remove dead cells. To test cellular uptake of antigen by BMDCs, RecE-Alexa Fluor647 was mixed with particles in H_2_O and incubated at room temperature for 15 min; complete growth medium (RPMI1640 with 10% FBS) was added. Day 6 purified BMDCs were dosed with samples for 24 h at 37°C. Cells were washed twice with DPBS, fixed with 4% paraformaldehyde for 10 min at room temperature, then stained with 30 µM DAPI for 20 min at room temperature. Cells were washed, mounted with Fluorsave (EMD MILLIPORE), and examined with a Zeiss 710 confocal microscope (Zeiss).
