# Supplementary figures and images for: Precisely Molded Nanoparticle Displaying DENV-E Proteins Induces Robust Serotype-Specific Neutralizing Antibody Responses
Source: PLoS Negl Trop Dis. 2016 Oct 20;10(10):e0005071. doi: 10.1371/journal.pntd.0005071 (PMC5072622; doi:10.1371/journal.pntd.0005071)

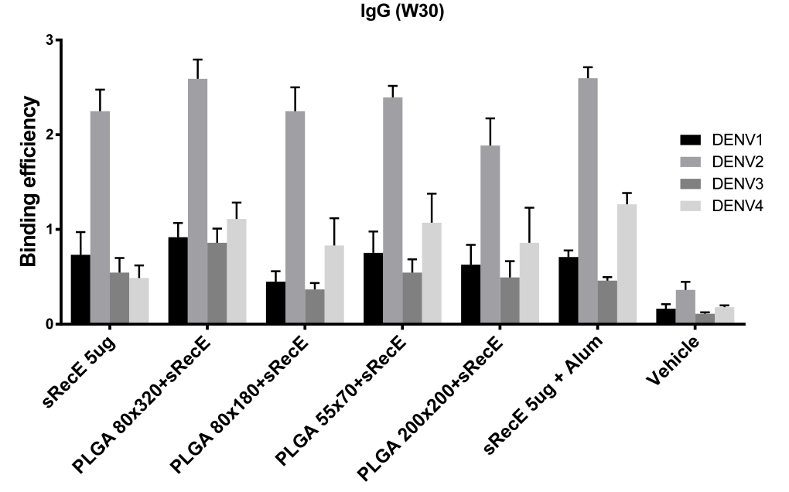

Supplement: S1 Fig — The capability of the cross reactive antibodies (at week 30 post immunization) to detect DENV1, DENV2, DENV3 or DENV4 was tested by a DENV specific capture ELISA. (TIF) [file pntd.0005071.s002.tif]

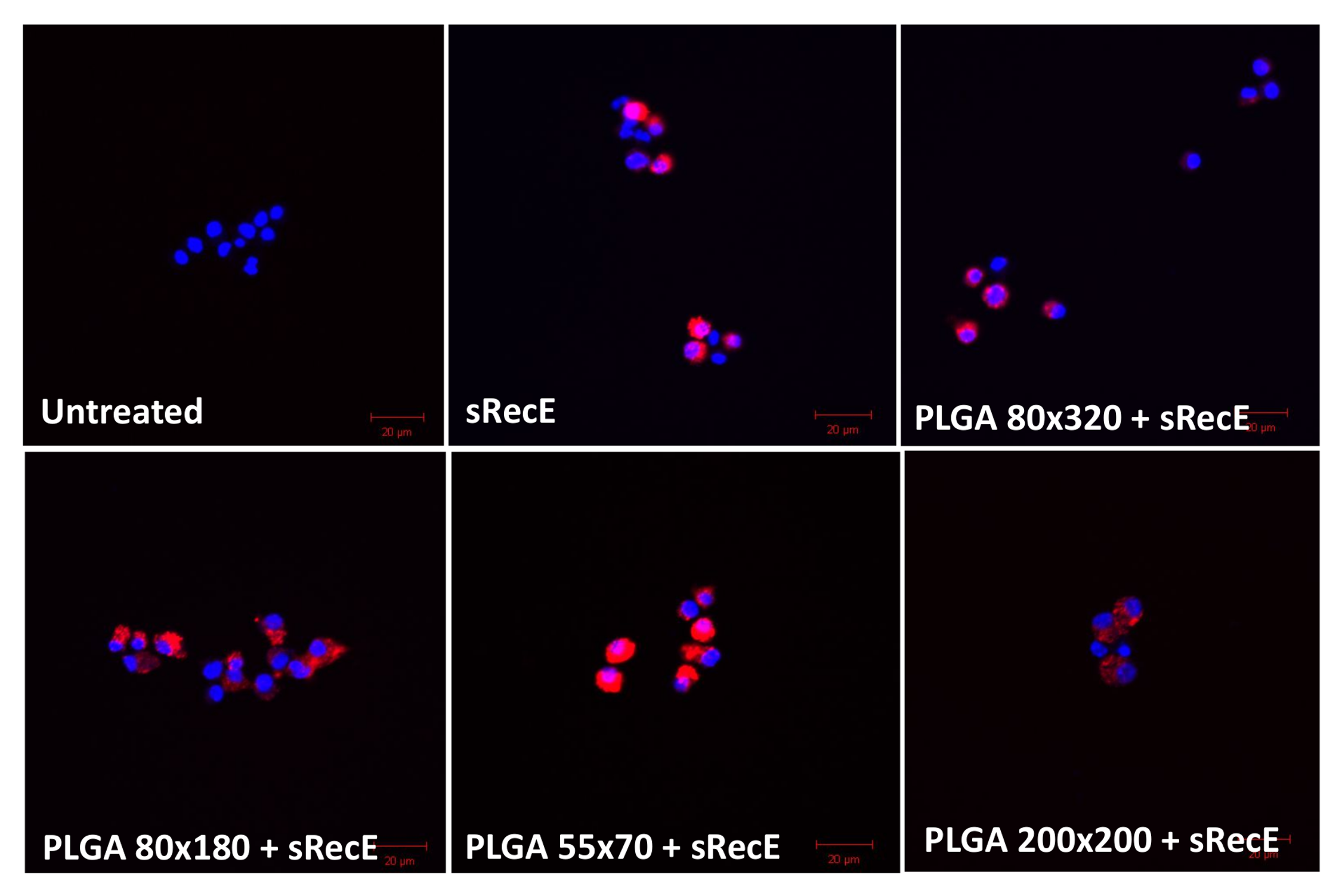

Supplement: S2 Fig — sRecE was tagged with Alexa Fluor 647 and adsorbed to PLGA nanoparticles. Bone marrow derived dendritic cells were mixed with the particles and analyzed with fluorescence microscopy to determine antigen uptake. (TIF) [file pntd.0005071.s003.tif]
